# Endorsement of International Consensus Radiochemistry Nomenclature Guidelines

**DOI:** 10.1186/s40658-018-0237-4

**Published:** 2019-04-16

**Authors:** Philip Elsinga, Angelika Bischof Delaloye, Arturo Chiti, Stefaan Vandenberghe, Francesco Giammarile, Ignasi Carrio

**Affiliations:** 1 Department of Nuclear Medicine and Molecular Imaging, University Medical Center Groningen, University of Groningen, Hanzeplein 1, 9713 EZ Groningen, The Netherlands; 2 Nuclear Medicine Service, Centre Hospitalier Universitaire Vaudois, Bugnon 46, 1011 Lausanne, Switzerland; 3 Department of Biomedical Sciences, Humanitas University, Milan, Italy; 4 Department of Diagnostic Imaging, Humanitas Clinical and Research Center – IRCCS, Milan, Italy; 5 MEDISIP-ELIS-IBITECH, Ghent University, Corneel Heymanslaan 10, ,9000 Gent, Belgium; 6 Nuclear Medicine and Diagnostic Imaging Section, Division of Human Health, International Atomic Energy Agency, Vienna, Austria; 7 Department of Radiology and Medical Physics, Hospital de Sant Pau, Barcelona, Spain

With the open letter published in *EJNMMI Radiopharmacy and Chemistry* by an international working group of experts, the EJNMMI journal family endorses the application of the guidelines in contributions to the journals.

Attached to the letter by the working group is a three-page summary highlighting the most relevant issues used in the notation of radiopharmaceuticals and related terms.

The full paper with all recommendations is published in “Consensus nomenclature rules for radiopharmaceutical chemistry—setting the record straight, Coenen, H.H., Gee A.D. et al., *Nuclear Medicine and Biology*, Volume 55, v – xi (2017) (10.1016/j.nucmedbio.2017.09.004)”.

All Editors-in-Chiefs of the EJNMMI journals strongly recommend all manuscripts meet these guidelines upon submission. In addition, all reviewers will be asked to be aware of the guidelines and wherever possible to check if the guidelines are followed.

Editors-in-Chief,

Philip Elsinga

Angelika Bischof Delaloye

Arturo Chiti

Stefaan Vandenberghe

Francesco Giammarile

Ignasi Carrio

